# Immunogenicity of Matrix Protein 2 Ectodomain (M2e) Displayed on Nodavirus-like Particles as Avian Influenza Vaccine for Poultry

**DOI:** 10.3390/vaccines13070701

**Published:** 2025-06-27

**Authors:** Anis Suraya Mohamad Abir, Wen Siang Tan, Abdul Rahman Omar, Kok Lian Ho, Munir Iqbal, Abdul Razak Mariatulqabtiah

**Affiliations:** 1 Laboratory of Vaccine and Biomolecules, Institute of Bioscience, Universiti Putra Malaysia, Serdang 43400, Selangor, Malaysia; anissuraya16@gmail.com (A.S.M.A.); aro@upm.edu.my (A.R.O.); 2 Department of Microbiology, Faculty of Biotechnology and Biomolecular Sciences, Universiti Putra Malaysia, Serdang 43400, Selangor, Malaysia; wstan@upm.edu.my; 3 Department of Veterinary Pathology and Microbiology, Faculty of Veterinary Medicine, Universiti Putra Malaysia, Serdang 43400, Selangor, Malaysia; 4 Department of Pathology, Faculty of Medicine and Health Sciences, Universiti Putra Malaysia, Serdang 43400, Selangor, Malaysia; klho@upm.edu.my; 5 Avian Influenza and Newcastle Disease Group, The Pirbright Institute, Woking GU24 0NF, UK; munir.iqbal@pirbright.ac.uk; 6 Department of Cell and Molecular Biology, Faculty of Biotechnology and Biomolecular Sciences, Universiti Putra Malaysia, Serdang 43400, Selangor, Malaysia

**Keywords:** avian influenza, nodavirus, M2e, VLP, vaccine

## Abstract

Avian influenza is an economically significant disease affecting poultry worldwide and is caused by influenza A viruses that can range from low to highly pathogenic strains. These viruses primarily target the respiratory, digestive, and nervous systems of birds, leading to severe outbreaks that threaten poultry production and pose zoonotic risks. The ectodomain of the avian influenza virus (AIV) matrix protein 2 (M2e), known for its high conservation across influenza strains, has emerged as a promising candidate for developing a universal influenza vaccine in a mouse model. However, the efficacy of such expression against poultry AIVs remains limited. The objective of this study was to evaluate the immunogenicity of nodavirus-like particles displaying the M2e proteins. In this study, three synthetic heterologous M2e genes originated from AIV strains H5N1, H9N2 and H5N2 were fused with the nodavirus capsid protein (NVC) of the giant freshwater prawn *Macrobrachium rosenbergii* (NVC-3xAvM2e) prior to immunogenicity characterisations in chickens. The expression vector pTRcHis-TARNA2 carrying the *NVC-3xAvM2e* gene cassette was introduced into *E. coli* TOP-10 cells. The recombinant proteins were purified, inoculated into one-week-old specific pathogen-free chickens subcutaneously and analysed. The recombinant protein NVC-3xAvM2e formed virus-like particles (VLPs) of approximately 25 nm in diameter when observed under a transmission electron microscope. Dynamic light scattering (DLS) analysis revealed that the VLPs have a polydispersity index (PDI) of 0.198. A direct ELISA upon animal experiments showed that M2e-specific antibodies were significantly increased in vaccinated chickens after the booster, with H5N1 M2e peptides having the highest mean absorbance value when compared with those of H9N2 and H5N2. A challenge study using low pathogenic AIV (LPAI) strain *A/chicken/Malaysia/UPM994/2018* (H9N2) at 10^6.5^ EID_50_ showed significant viral load in the lung and cloaca, but not in the oropharyngeal of vaccinated animals when compared with the unvaccinated control group. Collectively, this study suggests that nodavirus-like particles displaying three heterologous M2e have the potential to provide protection against LPAI H9N2 in chickens, though the vaccine’s efficacy and cross-protection across different haemagglutinin (HA) subtypes should be further evaluated.

## 1. Introduction

Avian influenza (AI) is a devastating poultry disease with serious economic consequences for the global poultry industry. Based on pathological effects, AI viruses (AIV) can be categorized into low pathogenic avian influenza (LPAI) and high pathogenic avian influenza (HPAI). This classification is based on the molecular characteristics and the ability of the viruses to cause varying degrees of disease [1]. HPAI viruses found in nature are mostly limited to the H5 and H7 subtypes, and infection with these viruses can result in up to 100% mortality in susceptible poultry species [2]. It is generally accepted that all H5 and H7 HPAI viruses originate from LPAI virus precursors through the insertion of multiple amino acids at the hemagglutinin (HA) cleavage site, caused by transcription errors, after the viruses have been introduced into the host [1]. These strains pose zoonotic health risks to humans. The recent H5N1 infections in dairy cows and poultry in the U.S. caused mild illnesses in patients with animal exposure, including conjunctivitis, fever and respiratory symptoms [3].

Vaccinations are recognised as a preventative measure against AIV in certain countries in the Asian and Middle Eastern regions, including Egypt, which have licensed more than 24 types of commercial H5 vaccines [4]. Oil-adjuvanted inactivated vaccines based on various H5 strains or classic H5N2 vaccine seed strains and live recombinant vaccines are the most typical vaccine platforms used [5]. Despite the demonstrated efficacy of these vaccines in reducing poultry mortality, clinical signs, shedding and transmission of the virus, their effectiveness is heavily reliant on antigenic matching with circulating viral strains, which influences the vaccine potency [6]. The challenges associated with individual administration and prior immunity, as well as vector-related issues, necessitate constant reformulation to match new field-circulating strains, which can present a significant challenge during an outbreak [6].

The design of a universal influenza vaccine has been a major focus in the field of influenza vaccinology as a new strategy to improve the performance of seasonal vaccines. Most attempts to create a universal influenza vaccine have been based on the external domain of the matrix 2 protein (M2e) which is more genetically conserved when compared with the haemagglutinin (HA) and neuraminidase (NA) surface glycoproteins of AIV [7]. Variations in M2e among influenza A virus strains can involve up to six amino acid differences, accounting for only 25% variation within the 23 amino acids of M2e [7]. First discovered in 1981, the M2 protein consists of 97 amino acid residues and is expressed from a separate open reading frame of segment 7 of the influenza viral genome. It projects on the surface of the virus as tetramers [8]. Structurally, M2 can be divided into three segments: the N-terminal ectodomain (positions 1–23), a transmembrane domain (positions 25–43), and an intracellular C terminal domain (positions 44–97) [9]. Among these segments, the N-terminal ectodomain is recognized by the host’s immune system, although it elicits a poor antibody response [10].

The M2e’s naturally low immunogenicity often necessitates coupling with various carriers to achieve optimal immunity [11]. With advancements in genetic engineering, larger carrier proteins, such as virus-like particles (VLPs), are frequently used to enhance immunogenicity [11]. VLPs mimic the natural structure of the native virus, yet do not consist of the complete virus genome. This renders them non-infectious, thus constituting a safer vaccine platform [11]. The hepatitis B core protein was the first VLP reported to successfully present exogenous M2e on its surface, providing protection in immunised mice upon lethal challenge [12]. Subsequently, studies have shown that multiple M2e antigens presented on nodavirus VLPs of the giant freshwater prawn species *Macrobrachium rosenbergii*, either in the presence or absence of an adjuvant, can induce strong M2e-specific antibody titres [13,14]. A significant protection against lethal challenges using H1N1 and H3N2 influenza A virus in mice has also been observed [14]. Furthermore, nodavirus VLPs displaying both avian and human influenza A virus M2e epitopes have been found to augment the activation of T helper cells, cytotoxic T lymphocytes and macrophages in chickens [15].

In the present study, we fused three heterologous M2e antigens from the H9N2, H5N2 and H5N1 AIV strains with the nodavirus capsid protein (NVC) to yield recombinant protein NVC-3xAvM2e. This recombinant protein formed VLPs which were inoculated into chickens to study their immunogenicity and protective efficacy upon challenge with LPAI H9N2. We demonstrated that NVC-3xAvM2e VLPs exhibited relatively strong immunogenicity, stimulated high titres of anti-M2e antibody, and showed low viral copy numbers in challenged chickens, suggesting limited viral replication.

## 2. Materials and Methods

### 2.1. Ethical and Biosafety Clearance

All procedures were approved by the Institutional Animal Care and Use Committee, Universiti Putra Malaysia (reference numbers UPM/IACUC/AUP-R065/2019 and UPM/IACUC/AUP-R054/2020), and the Department of Biosafety Malaysia (reference numbers JBK(S) 600-3/1/20 and JBK(S) 600-3/1/68).

### 2.2. Plasmids and Antigens

The expression vector pTrcHis-TARNA2 harbouring the *RNA2 capsid* gene of the *Macrobrachium rosenbergii* nodavirus capsid protein (NVC), with accession number AHM92901.1 [15], was obtained from the Molecular Virology Laboratory, Faculty of Biotechnology and Biomolecular Science, Universiti Putra Malaysia. Genes encoding three heterologous M2e from different AIV strains, namely *A/chicken/Vietnam/HU1-786/2014 (H9N2)*, *A/chicken/Wuhan/WHJF/2014 (H5N2)* and *A/chicken/Thailand/04 (H5N1)*, were synthetically synthesised and inserted into plasmid pUCIDT-Amp (Integrated DNA technologies (IDT), Coralville, IA, USA).

### 2.3. Cloning and Transformation of Plasmid Encoding NVC-3xAvM2e

Plasmid pTrcHis-TARNA2 was extracted using QIAprep Spin Miniprep kit (Qiagen, Hilden, Germany), according to the manufacturer’s instruction. The extracted pTrcHis-TARNA2 and pUCIDT-Amp carrying three heterologous *M2e* genes (3xAvM2e) were separately digested using *Eco*RI HF and *Hin*dIII HF (New England Lab, Woburn, MA, USA) for 1 h at 37 °C and heat inactivated at 60 °C for 20 min. The digested products were separated using 1% (*w*/*v*) agarose gel electrophoresis and purified using QIAquick Gel Extraction Kit (Qiagen, Hilden, North Rhine-Westphalia, Germany). The purified 3xAvM2e DNA fragment was inserted into *Eco*RI and *Hin*dIII sites at the 3′ end of the *NVC* gene in the pTrcHis-TARNA2 with the ratio of 1:5 in a 15 μL mixture of buffer containing T4 DNA ligase (Thermo Fisher, Waltham, MA, USA) at 4 °C for 16 h. Then, the mixture was used in transformation in a freshly prepared solution of competent *E. coli* TOP10 cells (Thermo Fisher, USA) using the heat shock method. Selection of positive transformants was performed in the presence of 100 μg/mL of ampicillin on LB agar plates. The inserted *3xAvM2e* gene and its orientation in pTrcHis-TARNA2 were confirmed by analytical PCR, i.e., 15 μL total reaction containing 10 ng of purified plasmid, 1.25 U DreamTaq DNA polymerase (Thermo Fisher, USA), 1X DreamTaq Buffer, 0.2 μM dNTPs, 0.1 μM forward primer (5′-CGATTATAAGGGTGTGACTGACCC-3′) and reverse primer (5′-GCTGAAAATCTTCTCTCATCCGCC-3′), with the following reaction conditionsl: initial denaturation (94 °C for 1 min), denaturation (94 °C for 30 s, and 40 cycles), annealing (53 °C for 1 min) and extension (72 °C for 40 s), and DNA sequencing analysis using the aforementioned primers (Apical Scientific Sdn Bhd., Seri Kembangan, Selangor, Malaysia).

### 2.4. SDS-PAGE and Western Blotting

A single bacterial colony harbouring the coding region NVC-3xAvM2e was inoculated into 50 mL of LB broth containing 100 μg/mL ampicillin and incubated overnight at 37 °C with shaking at 220 rpm. A 10 mL aliquot of the overnight culture was further grown until an OD_600_ of 0.6–0.8 was reached, prior to protein induction with 1 mM IPTG. The collected pellets were resuspended in 4X loading dye [10 µL; 100 mM Tris-HCl (pH 6.8), 20% (*v*/*v*) glycerol, 4% (*w*/*v*) SDS, 0.2% (*w*/*v*) bromophenol blue, 50 mM dithiothreitol] and boiled for 10 min. The samples were then loaded onto a 12% (*w*/*v*) SDS-polyacrylamide gel (PAGE) prepared using the Mini-PROTEAN 3 system (Bio-Rad, Hercules, CA, USA) and electrophoresed with running buffer [25 mM Tris-aminomethane, 192 mM glycine, 0.1% (*w*/*v*) SDS] at 16 mA for 70 min. PM2510 ExcelBand™ Enhanced 3-color Regular Range Protein Marker was included (SMOBIO Technology, Inc., Baoshan Township, Hsinchu City, Taiwan). The gel was subsequently stained with Coomassie Brilliant Blue staining solution [0.1% (*w*/*v*) Coomassie Brilliant Blue R-250 (Thermo Fisher, USA), 40% (*v*/*v*) methanol, 10% (*v*/*v*) acetic acid] for 20 min at room temperature with gentle shaking. Excess stain was removed using a de-staining solution [10% (*v*/*v*) methanol, 10% (*v*/*v*) acetic acid] for 1 h.

An unstained, replicate SDS-PAGE gel containing the protein samples was electroblotted onto nitrocellulose membranes (Pall Corporation, Portsmouth, Hampsire, UK) using a semi-dry Western blot apparatus (Bio-Rad, Hercules, CA, USA). The reaction was mediated by a transfer buffer [25 mM Tris-HCI, 194 mM glycine, 20% (*v*/*v*) methanol; pH 8.3] for 50 min at 24 V. Then, the nitrocellulose membrane was rinsed with distilled water and blocked with skimmed milk buffer [10% (*w*/*v*) Anlene brand of Fonterra, Auckland Central Business District, Auckland, New Zealand] prepared in TBS [50 mM Tris-HCI, 150 mM NaCl; pH 7.4] for 2 h at room temperature with gentle shaking. The membrane was washed three times with 1X TBST buffer [50 mM Tris-HCI, 150 mM NaCl, 0.05% Tween-20; pH 7.4] for 10 min per wash, followed by incubation with anti-His monoclonal antibody (1:3000 dilution in TBS; Merck, Rahway, NJ, USA) at 4 °C with gentle shaking for 2 h. The membrane was then washed three more times with 1X TBST buffer, followed by incubation with an alkaline phosphatase (AP)-conjugated anti-mouse IgG antibody (1:5000 dilution in TBS; Merck, USA) for 1 h with gentle shaking. After incubation, the membrane was washed three times with 1X TBST buffer and subjected to colour development using 5-bromo-4-chloro-3′-indolyl phosphate p-toluidine salt/nitro-blue tetrazolium chloride (BCIP/NBT) prepared in alkaline phosphatase buffer (100 mM Tris-HCl, 100 mM NaCl, 5 mM MgCl_2_; pH 9.5).

### 2.5. Time Point and Solubility Analysis of the Recombinant Proteins

Time point analysis of the NVC-3xAvM2e recombinant protein expressions post-induction with IPTG within 12 h was performed according to the established method [15]. Another batch of *E. coli* TOP10 cell harbouring the pTrcHis-TARNA with the *NVC-3xAvM2e* gene cassette was induced with 1 mM IPTG and incubated at three different temperatures, 18 °C, 25 °C and 37 °C at 220 rpm. At 5 h post-induction, the cultures were pelleted down by centrifugation at 8000× *g* for 10 min at 4 °C. The supernatant was carefully discarded, and the pellet was resuspended in 15 mL of lysis buffer [25 mM HEPES, 500 mM NaCl; pH 7.4]. Subsequently, 20 μg/mL DNase I, 4 mM of MgCl2, 0.2 mg/mL of lysozyme and 2 mM phenylmethylsulfonyl fluoride (PMSF) were added to the mixture followed by incubation at room temperature for 2 h on a disc rotor. The suspension was then sonicated at 24 MHz for 20 cycles, with 30 s cooling intervals on ice. The cell lysate was centrifuged at 8000× *g* for 15 min at 4 °C. The supernatant and pellet were retained as the soluble and insoluble fractions, respectively. Equal aliquots of total, soluble, and insoluble proteins were subjected to SDS-PAGE and western blot analysis.

### 2.6. Purification of NVC-3xAvM2e

A starter culture carrying the *NVC-3xAvM2e* gene cassette was induced with 1 mM IPTG and incubated at 25 °C with agitation at 220 rpm for 5 h. The cultures were then harvested and lysed for 2 h at room temperature. Following that, the cells were sonicated at 24 MHz on ice for 30 s, with 20 s breaks, for a total of 30 cycles. The cell supernatant was recovered by centrifugation at 8000× *g* for 15 min at 4 °C. After centrifugation, the supernatant was collected and filtered through a 0.45 μM syringe filter (Pall Corporation, Portsmouth, Hampshire, UK) prior to purification via a 1 mL HisTrap HP affinity column, a high-performance immobilized metal affinity chromatography (IMAC) (GE Healthcare, Chicago, IL, USA). The column was pre-washed with five column volumes of distilled water before equilibration with five column volumes of binding buffer [25 mM HEPES, 500 mM NaCl; pH 7.4]. The protein was loaded into the column and washed with ten column volumes of washing buffer [25 mM HEPES, 500 mM NaCl; pH 7.4] containing 50 mM imidazole. The bound histidine-tagged protein was then eluted from the column with ten column volumes of elution buffer [25 mM HEPES, 500 mM NaCl; pH 7.4] containing a gradient of imidazole concentrations (100 mM, 200 mM, and 500 mM). The eluted fractions were then analysed using SDS-PAGE and western blotting.

### 2.7. Dynamic Light Scattering (DLS) Analysis

Dynamic light scattering was carried out using a Zetasizer Nano S instrument (Malvern Panalytical, Grovewood Road, Malvern, UK). Measurements were conducted at 25 °C in a buffer containing 25 mM HEPES and 500 mM NaCl at pH 7.4.

### 2.8. Transmission Electron Microscope

Purified NVC-3xAvM2e was diluted in phosphate-buffered saline to a final concentration of 50 μg/μL. A 5 μL aliquot of the protein sample (250 μg) was applied onto a prepared carbon-coated copper grid and allowed to adsorb for 2 min. The grid was then negatively stained using 5 μL 2% (*w*/*v*) uranyl acetate at room temperature for 5 min to enhance contrast. Any excess sample and stain were gently blotted away with filter paper. Once dried, the grid was visualized under a transmission electron microscope (JEM-3100F; JEOL, Tokyo, Japan). The diameter of the VLPs were measured using a browser-based online measurement tool, ImageMeasurement (https://imagemeasurement.online/image/select, accessed on 15 May 2024).

### 2.9. Animals and the Challenge Virus

Ten-day-old SPF eggs were obtained from the Malaysian Vaccine Pharmaceuticals Sdn. Bhd. (MVP), Puchong, Selangor, Malaysia. The eggs were hatched at the Laboratory of Vaccine and Biomolecules, Institute of Bioscience, Universiti Putra Malaysia. After hatching, the chicks were transferred to a BSL2-certified animal house under the same laboratory. The chicks were then randomly divided into groups and provided with a commercial diet (Gold Coin 201C; Gold Coin Feedmills, Pelabuhan Klang, Selangor, Malaysia) and free access to water. The AIV used for the challenge study was an LPAI, strain *A/chicken/Malaysia/UPM994/2018 (H9N2)*. The virus was propagated and titrated in 9-day-old embryonated specific pathogen-free (SPF) chicken eggs, and the EID_50_ measurement was performed.

### 2.10. Vaccination and Challenge Study in Chickens

Forty-eight SPF chickens were randomly assigned to four groups (*n* = 12). Group 1 received purified NVC-3xAvM2e (100 μL; 0.5 mg/mL), Group 2 received purified NVC (100 μL; 0.5 mg/mL), Group 3 received inactivated H9N2 virus strain, and Group 4 received phosphate-buffered saline (PBS). Before subcutaneous injection, the purified recombinant proteins were dialysed against HEPES buffer [25 mM HEPES, 500 mM NaCl; pH 7.4] in Slide-A-Lyzer™ Dialysis Cassettes (3.5 K MWCO; Thermo Fisher, Waltham, MA, USA) for 4 h, followed by overnight dialysis. The concentration of the proteins was determined with the Bradford assay. Then, the NVC-3xAvM2e vaccine was emulsified with CpG ODN 2007 adjuvant (0.5 mg/mL, 1:1 ratio) (InvivoGen, San Diego, CA, USA) [16], filtered through a syringe (0.45 µM; Pall Corporation, Portsmouth, Hampsire, UK) and administered intramuscularly. Immunisation began at one week old, followed by boosters two weeks apart. Exactly two weeks after the first booster, all chickens except the negative control group were challenged via eye drop with 0.1 mL of 10^6.5^ EID_50_/mL LPAI H9N2. Lung tissues, cloacal, and oropharyngeal swabs (*n* = 3) were collected on days 3, 5, and 7 post-infection (*N* = 9), placed in RNAlater (Ambion, Austin, TX, USA) prior to RNA extraction and qPCR analysis. Blood samples were collected weekly before the challenge via brachial wing vein. Sera were isolated by incubating samples at room temperature for 30 min, followed by centrifugation at 3500× *g* for 10 min, then stored at −80 °C.

### 2.11. Measurement of Anti-M2e Specific Antibodies Using ELISA

Chemically synthesized AIV M2e epitopes from H9N2 (SLLTEVETLTKTGWECNCSGSSD), H5N2 (SLLTEVETPTRNGWECKCSDSSD), and H5N1 (SLLTEVETPTRNEWECRCSDSS) were used to coat a flat-bottom 96-well ELISA plate at a concentration of 2 μg/mL each (GenScript Biotech Corporation, Piscataway, NJ, USA; 100 μL) and incubated overnight at 4 °C. Unbound antigens were discarded, and plates were rinsed with 1X TBST (50 mM Tris-HCl, 150 mM NaCl, 0.05% Tween-20; pH 7.4), then blocked with 1% Blocker™ Casein (Thermo Fisher, Waltham, MA, USA) at room temperature for 2 h with constant shaking. After incubation, plates were rinsed three times with 1X TBST and incubated with collected sera (1:2000 dilution in 1X TBS) for 1 h at room temperature. Sera from chickens vaccinated with PBS and NVC (without M2e) served as negative controls, while inactivated H9N2 virus was used as a positive control (*n* = 6). Following three washes with 1X TBST, detection was performed by adding horseradish peroxidase (HRP)-conjugated goat anti-chicken IgY (1:5000 dilution in 1X TBS; NovusBio, Littleton, CO, USA) to each well, followed by incubation for 1 h at room temperature with constant shaking. After rinsing, plates were developed using 1-Step™ Ultra tetramethylbenzidine (TMB) substrate (Thermo Fisher, Waltham, MA, USA) and stopped by adding 50 µL of 1 M sulfuric acid (H_2_SO_4_) after 20 min of incubation at room temperature. Absorbance at 450 nm was measured using an ELx800 microtiter plate reader (Bio-Tek Instruments, Winooski, VT, USA).

### 2.12. Measurement of Viral Load Using Quantitative RT-PCR

Viral RNA from chicken lungs (30 mg, disrupted using TissueRuptor II; Qiagen, Germany), cloacal swabs, and oropharyngeal swabs collected on days 3, 5, and 7 post-challenge was extracted using the RNeasy Plus Mini Kit (Qiagen, Hilden, North Rhine-Westphalia, Germany) following the manufacturer’s protocol. RNA concentration was standardised to 20 ng (1 µL) and subjected to quantitative reverse transcriptase PCR (qPCR) (Mastercycler Realplex; Eppendorf, Barkhausenweg, Hamburg, Germany) using the SensiFast Probe No-ROX One-Step Kit (Bioline, Memphis, TN, USA). The reaction mixture was prepared in a 0.2 mL tube strip in the dark. The qPCR reaction mix included 10 µL of 2× SensiFast Probe No-ROX One-Step Mix, 0.2 µL of reverse transcriptase, 0.4 µL of RiboSafe RNase inhibitor, 400 nM forward primer (5′-TGCAGCGTAGACGTTTTGTC-3′), 400 nM reverse primer (5′-CAAGCGCACCAGTTGAGTAA-3′), and 100 nM TaqMan probe (5′/56-FAM/-TAAATGGGAATGGAGACCCA/3IABkFQ/-3′). Thermal cycling conditions are as follows: reverse transcription at 45 °C for 10 min, followed by 40 cycles of polymerase activation at 95 °C for 2 min, denaturation at 95 °C for 5 s, and annealing/extension at 60 °C for 5 s. Fluorescence data were collected during the annealing/extension step. All qPCR reactions were performed in triplicate, with a non-template control included. Results were expressed as log_10_ at each time point. The absolute quantification of viral RNA copies in swabs or lungs was based on the quantification cycle (Ct) value from each sample and the qPCR standard curve [14].

### 2.13. Statistical Analysis

Immunogenicity and viral shedding data were analysed using one-way analysis of variance (ANOVA), followed by Duncan’s multiple range test in SPSS Statistics software Version 29 (IBM Corporation, New York, NY, USA). Differences with a *p*-value lower than 0.05 were considered statistically significant.

## 3. Results

### 3.1. Construction of Plasmid pNVC-3xAvM2e

**Figure 1:**
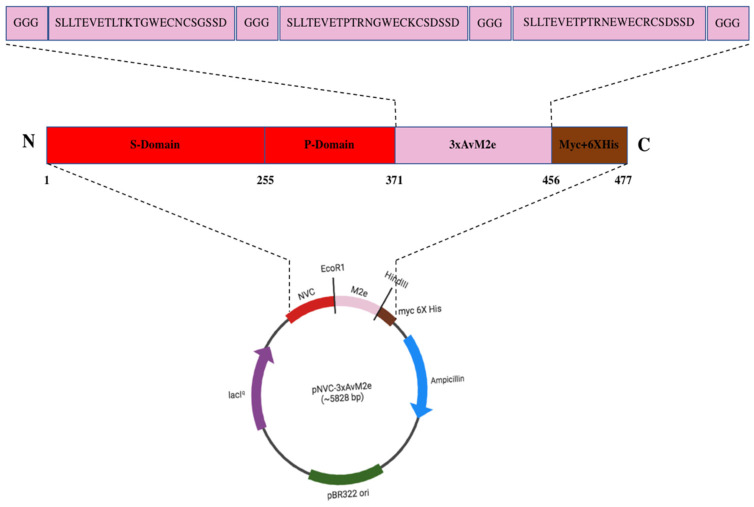
The pNVC-3xAvM2e construct. The insertion of the three heterologous *M2e* genes encoding SLLTEVETLTKTGWECNCSGSSD (H9N2), SLLTEVETPTRNGWECKCSDSSD (H5N2), and SLLTEVETPTRNEWECRCSDSSD (H5N1)) was performed at the carboxyl terminus of the NVC P-domain in pTcHis-TARNA2. A linker consisting of three glycine residues was added at both terminal ends and between each M2e peptide sequence.

Figure 1 illustrates the plasmid pNVC-3xAvM2e construct, where the insertion was performed at the carboxyl- terminus of the NVC P-domain in pTcHis-TARNA2. A linker consisting of three glycine residues was added at both terminal ends and between each M2e sequence.

### 3.2. Cloning and Expression of NVC-3xAvM2e

**Figure 2:**
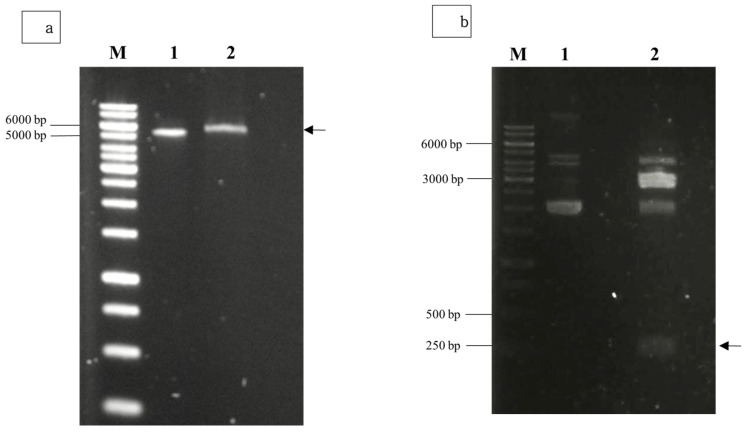
Restriction enzyme digestion using *Eco*RI and *Hin*dIII for (**a**) pTcHis-TARNA2 plasmid, and (**b**) pUCIDT-Amp plasmid carrying the *3xAvM2e* gene. Lane 1, undigested plasmids; lane 2, digested plasmids. Arrows indicate the sizes of the digested pTrcHis-TARNA2 (5600 bp) and the coding region of 3xAvM2e (260 bp). Lane M, DNA ladder marker (Promega, San Luis Obispo, CA, USA).

**Figure 3:**
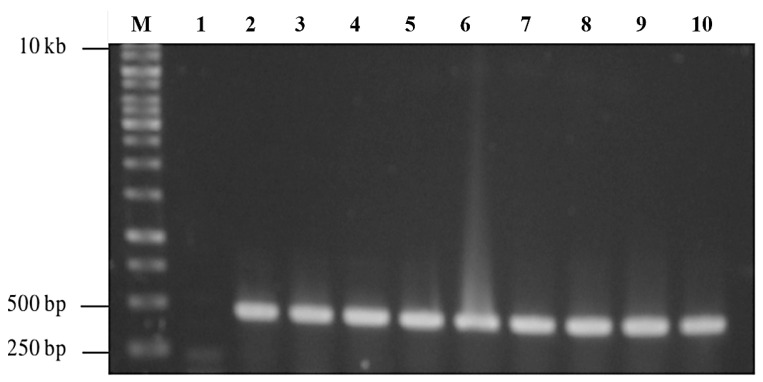
PCR screening of positive bacterial transformants carrying pTrcHis-TARNA2-3xAvM2e. Lane 1, negative control; lanes 2–10, PCR amplicons corresponding to the expected size of 453 bp; lane M, DNA ladder markers (Promega, San Luis Obispo, CA, USA).

**Figure 4:**
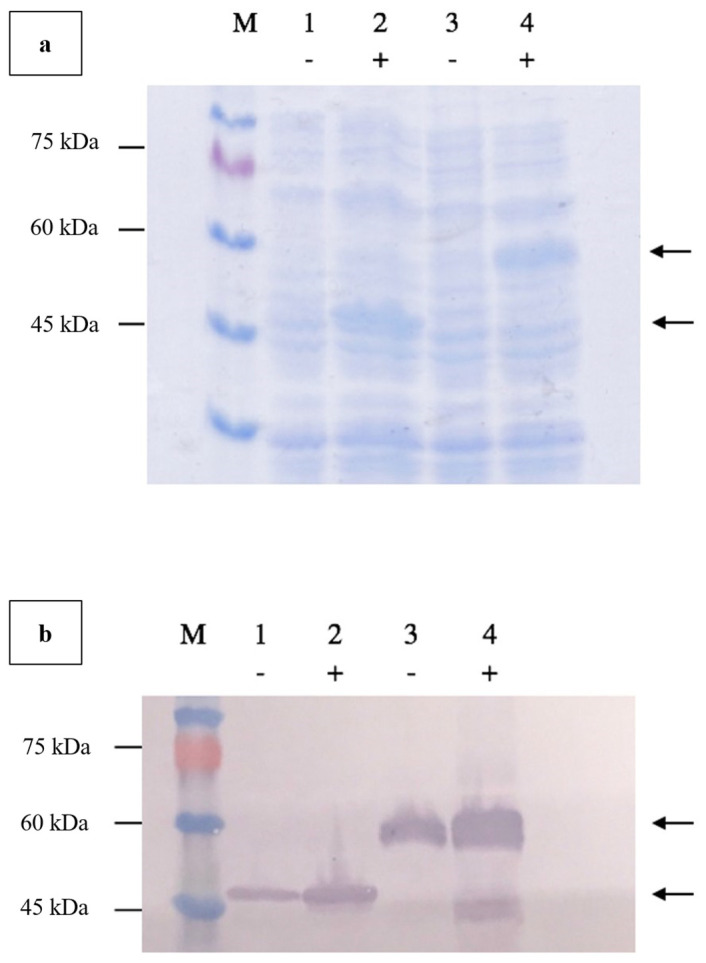
Verification of NVC-3xAvM2e protein expressed in *E. coli* using (**a**) SDS PAGE, and (**b**) western blotting using anti-His monoclonal antibodies. Lanes 1 and 2, NVC; lanes 3 and 4, NVC-3xAvM2e; lane M, PM2510 protein ladder marker (SMOBIO Technology Inc., Baoshan Township, Hsinchu City, Taiwan). Uninduced proteins are marked as (−) while IPTG-induced proteins are marked as (+). Arrows indicate the sizes of the expressed NVC (46 kDa), and NVC-3xAvM2e (53.9 kDa) proteins, respectively.

Agarose gel electrophoresis of the double-digested pTcHis-TARNA2 vector and *3xAvM2e* gene insert revealed bands of 5600 bp and 260 bp, corresponding to the predicted sizes (Figure 2). PCR confirmed the successful ligation of the digested *3xAvM2e* into the pTcHis-TARNA2 plasmid, producing the expected 453 bp amplicon (Figure 3). Sequencing analysis further validated the correct orientation of the *3xAvM2e* gene insertion between the *Eco*RI and *Hin*dIII restriction sites. The expression of NVC and recombinant NVC-3xAvM2e is regulated under the control of the *trc* promoter which produced translated proteins of 46 and 53.9 kDa, respectively, upon induction with IPTG (Figure 4a), and they were detected by an anti-His antibody in western blot analysis (Figure 4b). This result suggests that NVC-3xAvM2e was successfully expressed in *E. coli*, and its detection using the anti-His antibody indicates that the protein was completely synthesised, extending up to the His-tag.

### 3.3. Time Induction and Solubility Analysis

**Figure 5:**
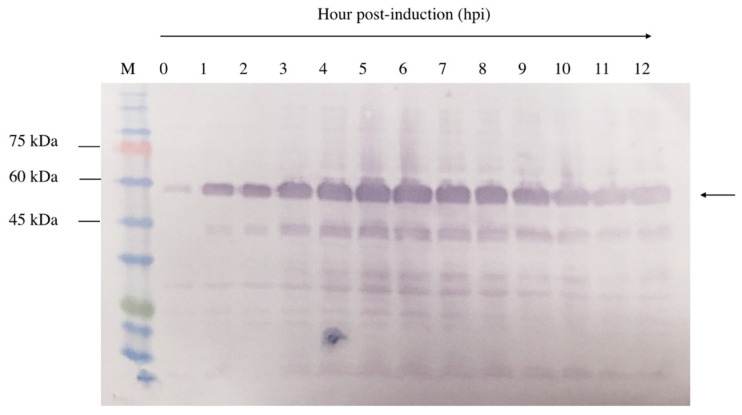
Time induction analysis of NVC-3xAvM2e expressed in *E. coli* cells after induction with IPTG. Lane 1, cell lysate before induction; lanes 2–12, cell lysates from the first to twelfth hours after IPTG induction; lane M, PM2510 protein ladder markers (SMOBIO Technology Inc., Baoshan Township, Hsinchu City, Taiwan). Arrow indicates the size of the expressed NVC-3xAvM2e at 53.9 kDa.

A 12 h time-course experiment was conducted to determine the optimal expression level of NVC-3xAvM2e in *E. coli* TOP10 cells. Upon IPTG induction, recombinant NVC-3xAvM2e expression was detected as early as 1 h post-induction, as evidenced by the appearance of a 53.9 kDa protein band (Figure 5). Over time, the band intensity gradually increased, indicating higher recombinant protein expression. Despite this, the 5 h post-induction time point was selected to ensure protein feasibility for downstream applications.

**Figure 6:**
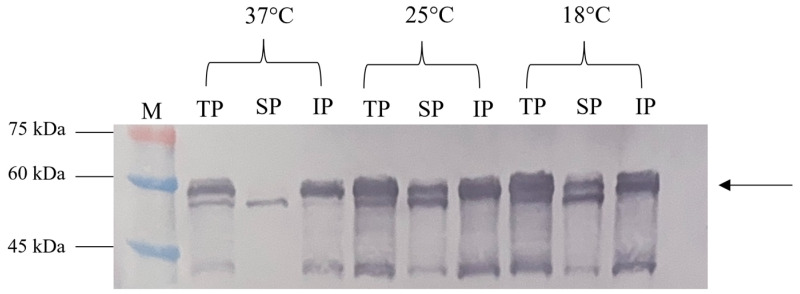
Effect of different incubation temperatures (37 °C, 25 °C and 18 °C) on the NVC-3xAvM2e protein’s solubility. TP, total protein; SP, soluble protein; IP, insoluble protein; lane M, PM2510 protein ladder marker (SMOBIO Technology Inc., Baoshan Township, Hsinchu City, Taiwan). Arrow indicates the size of the expressed NVC-3xAvM2e at 53.9 kDa.

The solubility assessment revealed that most NVC-3xAvM2e produced at 37 °C was in an insoluble form (Figure 6). However, at 25 °C and 18 °C, significantly higher levels of the soluble fraction were observed, consistent with the findings of [15].

### 3.4. Purification of NVC-3xAvM2e Protein

**Figure 7:**
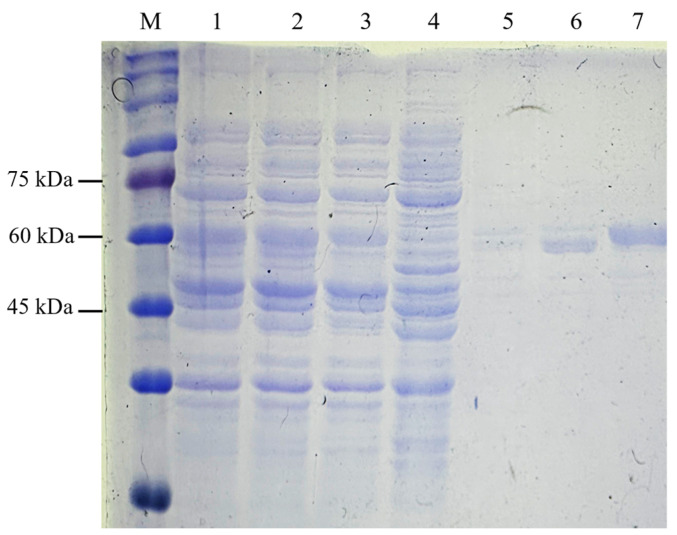
Purification of recombinant protein NVC-3xAvM2e using IMAC. Lane 1, supernatant; lane 2, filtered supernatant; lane 3, flow through; lanes 4–7, eluted sample after washing with lysis buffer containing 50 mM, 100 mM, 200 mM and 500 mM of imidazole, respectively; lane M, PM2510 protein ladder markers (SMOBIO Technology Inc., Baoshan Township, Hsinchu City, Taiwan).

The C-terminal His-tag on recombinant NVC-3xAvM2e facilitated purification via IMAC. Protein samples from the binding, washing, and elution steps were analysed using SDS-PAGE (Figure 7). NVC-3xAvM2e elution corresponded directly to the imidazole concentration. Most of the host proteins were removed during the washing stage, while complete elution of NVC-3xAvM2e was achieved at 500 mM imidazole buffer.

### 3.5. Characterization of the Recombinant Protein NVC-3xAvM2e

**Figure 8:**
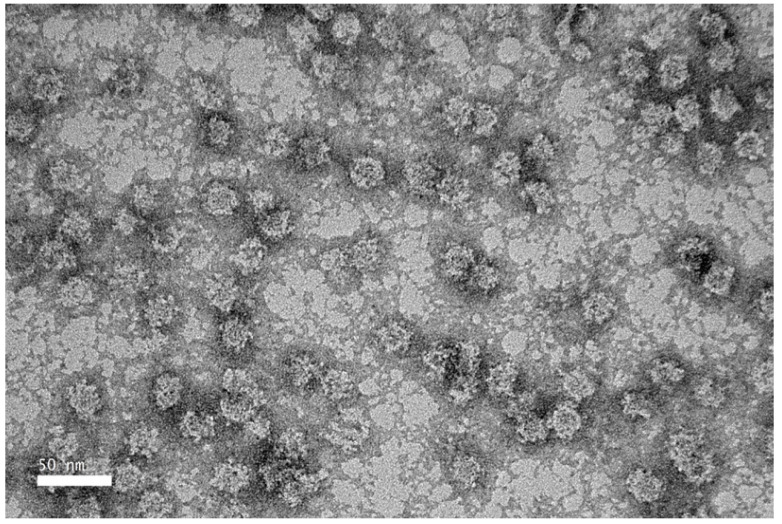
Transmission electron micrograph of purified NVC-3xAvM2e protein, negatively stained with 2% (*w*/*v*) uranyl acetate. The purified NVC-3xAvM2e (peptide concentration = 0.05 mg/mL) assembled into spherical VLPs with a diameter of ~25 nm.

**Figure 9:**
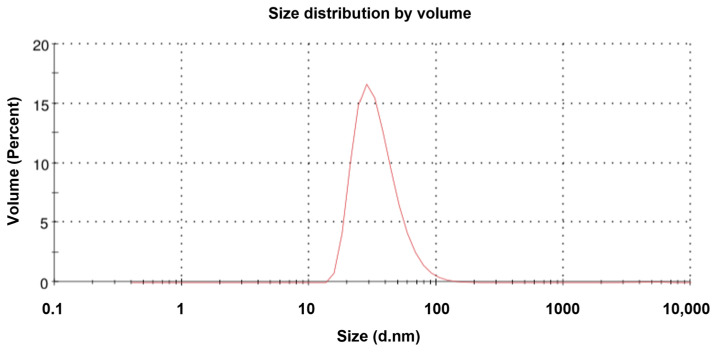
Dynamic light scattering analysis of the NVC-3xAvM2e proteins. The size distribution of the particles peaked at 35.4 nm.

TEM revealed that the purified NVC-3xAvM2e protein formed VLPs of ~25 nm in diameter (Figure 8), slightly smaller than the NVC VLPs (27 nm) [15]. The size observed under TEM was also smaller than that determined by DLS, which showed a hydrodynamic diameter peak at ~35.4 nm (Figure 9). Nevertheless, the NVC-3xAvM2e VLPs appeared intact and fairly monodisperse, consistent with DLS analysis, with a mean polydispersity index (PDI) of 0.198, indicating a homogeneous sample with uniformly sized particles.

### 3.6. Immunogenicity of the Chimeric VLPs in Chicken

**Figure 10:**
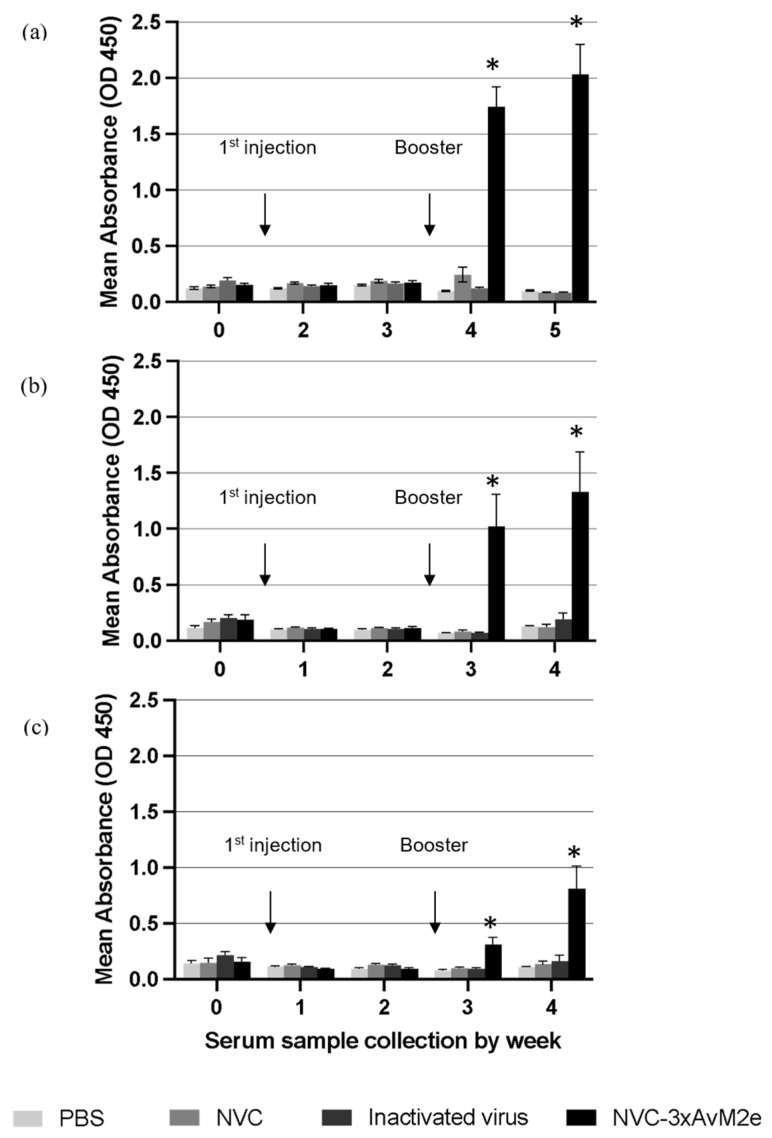
M2e-specific IgY antibody levels in chicken serum samples. Sera from chickens immunised with PBS, NVC, inactivated H9N2, and NVC-3xAvM2e were collected weekly prior to challenge, diluted at a 1:2000 ratio, and analysed by ELISA (*n* = 6). Antibody titres and immune responses were measured against synthetic M2e peptides of (**a**) H5N1, (**b**) H5N2, and (**c**) H9N2, which had been coated onto the ELISA plate. The reaction was detected using the goat anti-chicken IgY conjugated to horseradish peroxidase. An asterisk indicates statistical significance (*p* < 0.05) at each time point.

The anti-M2e immune response was monitored by determining the titre of M2e-specific immunoglobulin (IgY) in immunised chickens. No M2e-specific antibodies were detected before vaccination. A significant increase in M2e-specific antibody titres (*p* < 0.05) was observed following booster administration. Sera collected from NVC-3xAvM2e-immunized chickens exhibited reactivity with H5N1 (Figure 10a), H5N2 (Figure 10b), and H9N2 M2e peptides (Figure 10c) one week after booster administration. The M2e-specific antibody titres further increased two weeks after the booster. Although the elicited anti-M2e antibodies responded to all epitopes, sera incubated with the H9N2 M2e peptide exhibited lower M2e-specific antibody reactivity than those incubated with H5N1 and H5N2 M2e peptides after booster administration. Nevertheless, M2e-specific antibodies remained undetectable in sera from chickens immunised with inactivated H9N2 virus and control groups lacking the M2e epitopes, including PBS and NVC, throughout the immunisation period. The low immunogenicity of M2e in the context of inactivated H9N2 virus may be due to its relatively lower abundance in the virion compared with other viral proteins, such as HA and NA [17]. The reactivity of anti-M2e antibodies toward M2e peptides verifies that NVC-3xAvM2e is immunogenic.

### 3.7. Determination of Viral Load

**Figure 11:**
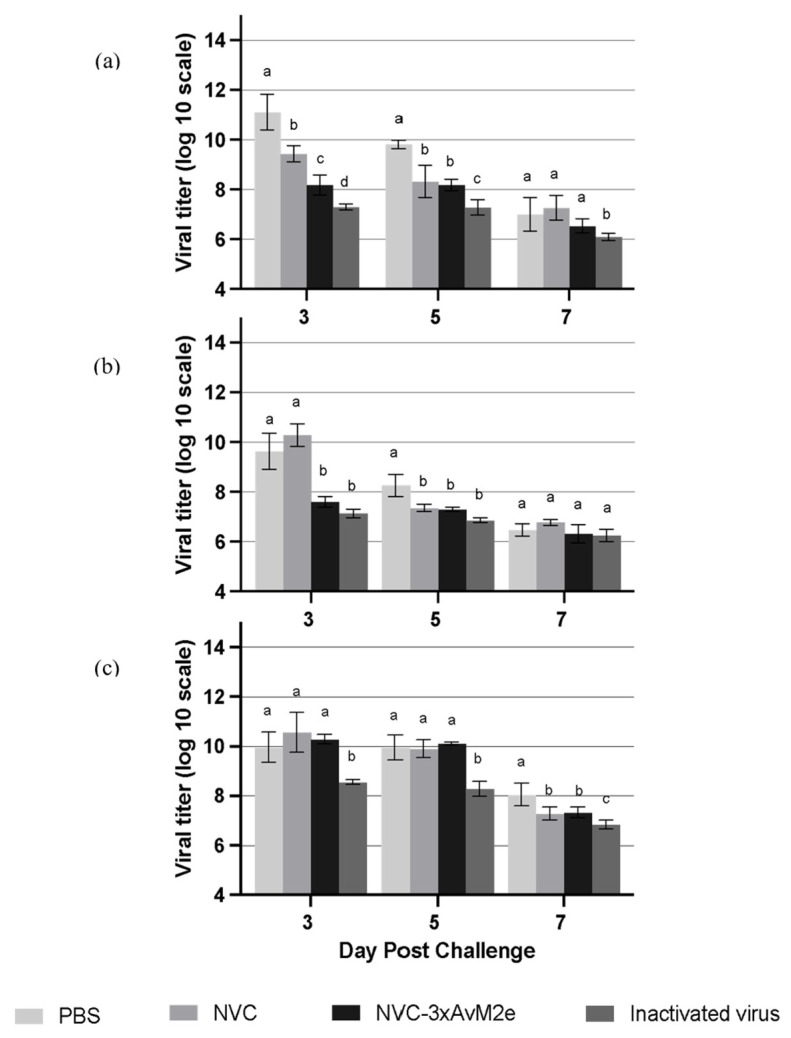
Viral tires of LPAI H9N2-challenged chickens (*n* = 3). Virus titres in the (**a**) lung, (**b**) cloacal swab, and (**c**) oropharyngeal swab were measured at days 3, 5, and 7 post-infection. Different letters denote statistically significant differences (*p* < 0.05) between groups, whereas shared letters indicate no significant differences within each time point. Error bars represent the mean ± standard error of triplicate measurements.

The protective efficacy of M2e-specific antibody responses induced by NVC-3xAvM2e was evaluated based on viral shedding reduction in individual chicken lungs (Figure 11a), cloacal swabs (Figure 11b) and oropharyngeal swabs (Figure 11c) on day days 3, 5 and 7 following a challenge with LPAI H9N2. On day 3 post-challenge, a significant reduction (*p* < 0.05) in viral shedding was observed in the lungs and cloacal samples of NVC-3xAvM2e-immunized chickens compared with those inoculated with PBS and NVC. By day 5, the PBS-immunised group continued to exhibit the highest viral loads in the lung and cloacal samples. However, no significant reduction in oropharyngeal viral loads was detected in the NVC-3xAvM2e-immunised group from days 3 to 5 post-challenge. Notably, chickens immunised with inactivated virus exhibited a lower reduction in viral titres between days 3 and 5 post-challenge compared with those inoculated with NVC-3xAvM2e.

## 4. Discussion

Currently, various commercial avian influenza vaccines are designed to be effective against matching vaccine strains but fail to induce cross-protective immune responses against constantly emerging antigenic variants of influenza A virus (IAV) [18]. As a result, developing a universal influenza vaccine has become a major focus in influenza vaccinology to provide broader protection against diverse IAVs. Instead of targeting the highly variable HA glycoprotein, conserved regions such as M2e have gained attention for universal vaccine development [19]. However, M2e has inherently low immunogenicity due to its small size, necessitating a larger carrier protein to enhance its immune response.

The number of M2e copies fused to the C-terminal end of NVC correlated with a greater induction of total antibodies against M2e [13], hence three heterologous *M2e* genes were used in the present study. The P-domain of NVC has been identified as a favourable site for peptide insertion on the outer surface of the VLPs formed by the NVC [20,21]. Previous studies have demonstrated that peptide insertion at the P-domain, particularly at the C-terminus of NVC, resulted in epitope expression on the VLP surface, which was highly immunogenic in mice [13,14]. The placement of peptides in NVC VLPs influences epitope conformation and its binding ability to host cell receptors. Additionally, glycine linkers reduce structural stiffness, allowing greater flexibility for adjacent protein domains to fold independently, and be freely displayed on the VLP surface [22].

We found that the expression of recombinant protein NVC-3xAvM2e peaked at the 5th hour post-IPTG induction and remained detectable up to the 12th hour. However, prolonged incubation led to increased medium acidity [23], promoting protein proteolysis and reducing overall yield. Therefore, the 5th hour post-induction was selected for downstream applications. Protein solubility was also identified as a critical factor for purification. In *E. coli*, inclusion bodies—insoluble aggregates of misfolded protein—often reduce solubility, necessitating the optimization of expression conditions. To enhance solubility, we tested three culture temperatures (18 °C, 25 °C, and 37 °C). Despite higher solubility at lower temperatures, 25 °C was chosen as the optimal condition, as it closely mimics the native propagation temperature of nodavirus in freshwater prawns [24].

IMAC, a widely used method for purifying His-tagged proteins, was employed to purify recombinant protein NVC-3xAvM2e. Most of the host proteins were efficiently removed with 50 mM imidazole, and the target protein was subsequently eluted with 500 mM imidazole. TEM confirmed that the purified NVC-3xAvM2e assembled into spherical VLPs when expressed in the *E. coli* system, closely resembling nodavirus VLPs. However, the fusion of three heterologous M2e antigens (~120 amino acid residues) on NVC resulted in a 2–5 nm reduction in VLP diameter when compared with that of native NVC [15]. Our results correspond to the study by Yong et al. (2015) [13] who found that adding 158 amino acid residues at the C-terminal end had no effect on VLP formation. We believed that the observed diameter reduction may be due to conformational disturbances in the P-domain of NVC, which accommodates the large protein fragment. Notably, in native NVC, the protruding P-domain contributes an additional 4.7 nm to the overall VLP diameter [20]. Despite this size reduction, DLS analysis indicated that NVC-3xAvM2e assembled into homogeneous VLPs with an average hydrodynamic diameter of 35.42 nm, larger than the TEM-measured diameter. This discrepancy may be attributed to ion interactions in solution via van der Waals forces, which contribute to an increased particle size in DLS analysis [25]. Additionally, TEM measurements were performed on a dried carbon grid, which may have influenced particle formation. A more advanced approach to strengthen the evidence of M2e surface display would be the use of atomic force microscopy and/or cryo-EM, which can provide nanometer-scale surface topology and high-resolution structural visualisation, respectively [20,26].

As antibodies serve as a crucial line of defence and a key indicator for evaluating vaccine-induced immunity, we sought to investigate the immunogenic properties of M2e displayed on nodavirus VLPs by immunising chickens with recombinant NVC-3xAvM2e. Our results demonstrate that the recombinant NVC-3xAvM2e successfully elicited a robust immune response, leading to a high level of anti-M2e IgY production post-immunisation. Sera collected from NVC-3xAvM2e-immunised chickens exhibited significant reactivity of anti-M2e IgY titers against H5N1, H5N2, and H9N2 M2e peptides following the booster immunisation. This finding aligns with a previous study [15], which reported a marked increase in anti-M2e antibody titres after secondary immunisation in chickens, both with and without adjuvants. The observed late-phase immune response can be attributed to the activation of naïve B cells upon antigen exposure, leading to their rapid expansion and differentiation into immunoglobulin-producing plasma cells. This, in turn, results in a heightened IgY response following the booster immunisation. These findings suggest that M2e displayed on NVC VLP serves as a strong immunogen capable of stimulating robust humoral immune responses.

Despite the overall strong immune response, sera incubated with H9N2 M2e peptide exhibited comparatively lower M2e-specific antibody reactivity. We hypothesize that this reduced reactivity may be due to the conformation in which the epitope is presented on the VLP surface. However, a molecular resolution imaging tool should be employed to visualize the epitope presentation and validate this hypothesis. It is possible that the epitope is partially shielded by surrounding structural elements, limiting its accessibility to humoral immune recognition, and thereby reducing antibody production. Additionally, the presence of cysteine residues in the three heterologous M2e sequences displayed on the NVC VLPs may have facilitated the formation of disulphide bridges, potentially leading to protein aggregation. This phenomenon could further disrupt epitope presentation, thereby affecting immune recognition and response [27,28]. Experimental validation should be performed to confirm these effects specifically for our vaccine construct.

Previous studies have demonstrated that anti-M2e antibodies can bind strongly to M2e on the surface of infected cells, thereby inhibiting viral replication [29,30]. As LPAI AIV typically induces mild clinical signs and does not result in mortality, unlike highly pathogenic AIV strains, protective efficacy could not be assessed based on percentage of animal survival. Instead, viral shedding reduction was used as a key indicator in this study to evaluate the extent of immune protection conferred by NVC-3xAvM2e immunisation. Our data revealed a significant reduction in viral load in the lungs and cloacal swabs of chickens immunised with NVC-3xAvM2e at the peak of LPAI H9N2 replication at day 3, when compared with the PBS and NVC vaccination groups. This suggests that immune responses triggered by NVC-3xAvM2e can effectively limit viral replication, although not completely. The reduced viral load in the lungs and cloaca, but not in oropharyngeal swabs, following NVC-3xAvM2e VLP immunization is likely due to differences in tissue tropism (i.e., the extent of viral replication and shedding can vary by tissue and route of infection) and local immune responses (i.e., the immune system may not have mounted a strong response in the oropharynx at the time of analysis) [31]. The viral shedding dynamics may also influence this, in which the oropharynx may remain a persistent site of viral replication due to its favourable microenvironment and less effective local immunity, while the lungs and cloaca are more effectively controlled by vaccine-induced immune responses (reviewed by [32]).

As anti-M2e antibodies are non-neutralizing [33,34], direct viral clearance was not expected. Despite their inability to neutralize the virus directly, anti-M2e antibodies may contribute to viral control by engaging immune mechanisms such as natural killer (NK) cell-mediated antibody-dependent cellular cytotoxicity (ADCC) [35]. This may explain the relatively weaker ability of anti-M2e antibodies to suppress viral replication. As anticipated, chickens immunised with whole inactivated H9N2 virus exhibited greater viral load reduction compared with those immunised with recombinant NVC-3xAvM2e, despite the latter induced high levels of M2e-specific antibodies. This observation is consistent with the findings by Elaish et al. (2017) [29], where chickens immunised with inactivated virus demonstrated greater viral neutralization and plaque reduction than those immunised with recombinant M2e displayed on the surface of norovirus P particles. This difference is likely due to the fact that antibodies generated by whole inactivated virus vaccines predominantly target the HA epitope, which is known for its strong neutralizing activity.

While the absence of cell-mediated immunity (CMI) data limits a full mechanistic understanding, the strong humoral immune responses, particularly the antigen-specific IgY antibody activity which was observed to be significantly increased in this study, serve as reliable indicators of AIV vaccine-induced protection in poultry [18]. Additionally, as the mechanism of action of M2e-based vaccines is primarily antibody-mediated, the inclusion of CMI assays in future studies is a progression rather than a prerequisite for initial validation of the NVC-3xAvM2e vaccine’s efficacy.

## 5. Conclusions

In summary, we successfully cloned and expressed NVC-3xAvM2e in *E. coli*. The recombinant protein was purified using IMAC and demonstrated the ability to self-assemble into VLPs, as verified by TEM and DLS analyses. The self-assembling nanoparticle displaying the antigenic M2e epitopes on its surface elicited strong humoral immune responses in chickens and significantly reduced viral replication following LPAI H9N2 challenge. These findings highlight the potential of nodavirus VLPs displaying heterologous M2e sequences as a promising vaccine candidate against LPAI H9N2 in poultry. Future studies should focus on evaluating cell-mediated immune responses against M2e, optimizing antigen presentation on the VLP surface, and assessing protective efficacy against multiple influenza virus strains to enhance the breadth of protection.

## Data Availability

Data will be made available on request.

## Abbreviations

The following abbreviations are used in this manuscript:

AIV	Avian influenza virus
M2e	Ectodomain of the influenza matrix protein 2
NVC	Nodavirus capsid protein RNA2
NVC-3xAvM2e	NVC originated from giant freshwater prawn *Macrobrachium rosenbergii* carrying three Copies of M2e genes
DLS	Dynamic light scattering
LPAI	Low pathogenic AIV
HPAI	High pathogenic AIV
HA	Haemagglutinin
NA	Neuraminidase
IMAC	Immobilized metal affinity chromatography
VLP	Virus-like particles
